# Emergence of linezolid-resistant *Enterococcus faecium* in a tertiary hospital in Copenhagen

**DOI:** 10.1099/mgen.0.001055

**Published:** 2023-07-06

**Authors:** Maria-Anna Misiakou, Frederik Boetius Hertz, Kristian Schønning, Susanne Häussler, Karen Leth Nielsen

**Affiliations:** ^1^​ Center for Genomic Medicine, Rigshospitalet, Copenhagen, Denmark; ^2^​ Department of Clinical Microbiology, Rigshospitalet, Copenhagen, Denmark; ^3^​ Department of Clinical Medicine, Faculty of Health and Medical Sciences, University of Copenhagen, Copenhagen, Denmark; ^4^​ Twincore, Centre for Experimental and Clinical Infection Research, Hannover, Germany

**Keywords:** selection pressure, oxazolidinone, VRE, outbreak, SNP, whole-genome sequencing, resistance mutation

## Abstract

Linezolid is used as first-line treatment of infections caused by vancomycin-resistant *Enterococcus faecium *. However, resistance to linezolid is increasingly detected. The aim of the present study was to elucidate the causes and mechanisms for the increase in linezolid-resistant *E. faecium* at Copenhagen University Hospital – Rigshospitalet. We therefore combined patient information on linezolid treatment with whole-genome sequencing data for vancomycin- or linezolid-resistant *E. faecium* isolates that had been systematically collected since 2014 (*n*=458). Whole-genome sequencing was performed for multilocus sequence typing (MLST), identification of linezolid resistance-conferring genes/mutations and determination of phylogenetically closely related strains. The collection of *E. faecium* isolates belonged to prevalent vancomycin-resistant MLST types. Among these, we identified clusters of closely related linezolid-resistant strains compatible with nosocomial transmission. We also identified linezolid-resistant enterococcus isolates not genetically closely related to other isolates compatible with *de novo* generation of linezolid resistance. Patients with the latter isolates were significantly more frequently exposed to linezolid treatment than patients with related linezolid-resistant enterococcus isolates. We also identified six patients who initially carried a vancomycin-resistant, linezolid-sensitive enterococcus, but from whom vancomycin-resistant, linezolid-resistant enterococci (LVRE) closely related to their initial isolate were recovered after linezolid treatment. Our data illustrate that linezolid resistance may develop in the individual patient subsequent to linezolid exposure and can be transmitted between patients in a hospital setting.

## Data Summary

Raw sequencing reads of 492 *E. faecium* isolates have been deposited at the European Nucleotide Archive under accession number PRJEB57197. The authors confirm all supporting data have been provided within the article or through supplementary data files.

**Impact Statement**

We show that linezolid treatment selects for linezolid-resistant *Enterococcus faecium* that harbour resistance-conferring mutations in individual patients. Furthermore, dissemination of linezolid-resistant isolates occurs in the hospital setting. This study highlights the possible consequences of linezolid use in a hospital setting with high prevalence of vancomycin-resistant isolates. Concurrent linezolid and vancomycin resistance in *E. faecium* further limits treatment options for these already difficult-to-treat infections.

## Introduction

Over the past two decades, increasing numbers of vancomycin-resistant enterococci (VRE), particularly *Enterococcus faecium *, have been reported in numerous healthcare facilities on a global scale. This is worrying, since *E. faecium* often are resistant to beta-lactam antibiotics, and so vancomycin is the first-line treatment [1]. VRE pose a significant antibiotic resistance threat, leading to 20 000 infections and 1300 deaths each year in the USA alone [2].

The capital region of Denmark has experienced multiple outbreaks with VRE carrying *vanA*, followed by an increase in *vanB*-carrying VRE over the last decade. Clonal shifts occur over time [3–6]. In Denmark, the prevalence of invasive (from normally sterile sites) vancomycin-resistant *E. faecium* infections has increased from 4 % in 2015 to 12 % in 2018 [7]. This is a much higher proportional increase than in the other Nordic countries, where the increase ranged between 0–2.3 % [7, 8], but lower than in Germany, where 14.9 % of clinical enterococcal infections are caused by VRE [9].

Linezolid is the first member of the oxazolidinone class of antimicrobials. It was approved in the USA for the treatment of nosocomial pneumonia and severe skin infections in the year 2000. Linezolid is highly efficient against Gram-positive cocci, including *Staphylococcus aureus* [methicillin-susceptible *S. aureus* (MSSA) and methicillin-resistant *S. aureus* (MRSA)], *Staphylococcus epidermidi* s and enterococci, and is used as a first-line treatment for VRE infections. At Copenhagen University Hospital, Rigshospitalet, linezolid is administered to patients infected by VRE as a monotherapy.

The resistance mechanism involves mutations in the loop of domain V in 23S or in ribosomal proteins L3, L4 and L22 to a smaller extent, inhibiting the binding of linezolid to the 23S ribosomal RNA of the 50S subunit and thereby preventing inhibition of the bacterial protein synthesis. The most common mutation is G2576T [10–12]. Additionally, resistance towards linezolid can be caused by the resistance genes *cfr*, *cfr(B*) and *cfr(D*), all encoding an rRNA methyltransferase causing multidrug resistance in the bacteria, as well as *optrA* and *poxtA* – both encoding an ABC-F family protein [13–15]. All of these five genes are often located on mobile genetic elements, such as plasmids or transposons, enabling horizontal transfer of resistance [16, 17]. In particular, *poxtA* has been described to possibly be linked to animal husbandry [16].

Originally, the emergence of linezolid-resistant strains was thought to be unlikely. Linezolid is a synthetic agent, with a low probability that natural resistance mechanisms are already present. Furthermore, oxazolidinones inhibit bacterial ribosomal protein synthesis by binding to the domain V of the 23S rRNA of the 50S ribosomal subunit, thus, blocking the formation of the initiation complex [18]. Since bacterial species often carry multiple copies of the 23S rRNA gene (four alleles in *E. faecalis *, five–six alleles in *E. faecium *), it was assumed that resistance would not readily arise, as this would require mutations in multiple 23S rRNA copies [19]. Finally, *in vitro* studies had shown that linezolid resistance rarely occurred [20].

The LEADER study has shown a linezolid resistance rate of <1 % between 2011–2015 and resistance was caused by *optrA, cfr* and 23S mutations [21]. Similarly, the European ZAAPS study identified >99.5 % susceptibility towards linezolid in *Enterococcus* spp. and *Staphylococcus* spp. [22]. Despite this, reports regarding occurrence and hospital outbreaks with linezolid-resistant enterococci (LRE) and staphylococci have been published [23, 24].

The aim of the present study was to use whole-genome sequencing data as well as patient data that have been collected over a period of more than 7 years to investigate whether the increasing prevalence of linezolid-resistant *E. faecium* in a large tertiary hospital is due to nosocomial transmission or emergence in individual patients subsequent to linezolid exposure.

## Methods

### Bacterial isolates

All *E. faecium* isolates that were included in this study were collected between 2014 and 2022. We collected and whole-genome sequenced *E. faecium* showing reduced susceptibility towards linezolid and/or vancomycin from clinical samples and rectal swabs (screening isolates) at Copenhagen University Hospital – Rigshospitalet, Denmark, *n*=492. Of these, 458 were individual patient samples excluding any isolates with identical genotypes [multilocus sequence typing (MLST) and VRE/LRE resistance mechanism] that were sampled multiple times (Tables 1, 2). These were identified in 447 patients, hence, patients were included more than once if the phenotype or genotype of the isolates changed. Longitudinal samples from the same patients were included in order to follow resistance development over time when this caused a change in genotype. Antimicrobial susceptibility testing was performed following European Committee on Antimicrobial Susceptibility Testing (EUCAST) guidelines for linezolid using antibiotic disc diffusion and with a breakpoint of ≥20 mm as susceptible according to EUCAST breakpoint table v. 12.0. We have applied the nomenclature of Bender *et al*. [17].

**Table 1.** Number of isolates in this study including vancomycin resistance genes and linezolid resistance

No. of isolates (*n*=458)	*vanA*	*vanB*	*vanA+vanB*	VSE
LRE (*n*=53)	12	22	0	19
LSE (*n*=405)	214	161	5	25

**Table 2.** Number of patients in this study and information on linezolid treatment

Patients	*n*	LZD treatment in relevant period
All^*^	**447**	**60**
**Patients with LRE**	**51**	**29**
Patients with VSE	19	14
Patients with VRE	32	15
**Patients with LSE**	**403**	**33**
Patients with VSE	25	0
Patients with VRE	378	33

*Patients with available treatment data. Two patients were sampled with both LSE and LRE.

### Data sources

All Danish citizens are assigned a unique and permanent civil registration number at birth and immigration. This enables individual-level linkage of local hospital as well as nationwide registers. This was used to obtain data on antibiotic treatment, which are available from hospital registers from 2017. For isolates collected prior to 2017 treatment was investigated manually for each individual patient. From the laboratory information system MADS (Mads-group, University Hospital Aarhus, Denmark), we obtained information on clinical isolates referred to the Department of Clinical Microbiology, including the results of phenotypic antibiotic susceptibility testing, which was available for the majority of the isolates (*n*=383).

### Patient linezolid treatment records

We identified linezolid exposure to patients based on prescriptions in the patient journal. We defined the relevant linezolid treatment period as at least 4 consecutive days of treatment and only considered samples taken between 4 days and up to 6 months after treatment start. The number of patients with relevant treatment in correlation to genotypic resistance is listed in Table 2. Only samples from patients with available treatment data were considered for subsequent analyses.

### Bacterial whole-genome sequencing

Genomic DNA was purified using DNeasy Blood and Tissue kit (Qiagen) and DNA libraries were constructed with a Nextera XT DNA Library Prep kit (Illumina). Three hundred and forty-seven libraries were sequenced on an Illumina MiSeq and 145 on a NextSeq 500 instrument, generating 250 or 150 base paired-end sequencing reads, respectively. A median of 1, 718, 611 paired-end reads and a median of 313 Mbp were generated across all isolates.

### Bioinformatics data processing and computational downstream analyses

Genomes for each isolate were processed using an in-house microbial genomics pipeline that includes steps for *de novo* assembly using shovill v.1.0.4 (https://github.com/tseemann/shovill), MLST using mlst v. 2.19.0 (https://github.com/tseemann/mlst) and prediction of linezolid and vancomycin resistance using LRE-Finder and abricate v.0.9.8 (https://github.com/tseemann/abricate) against the ResFinder database [25], respectively. The genomes from the entire collection were used to perform whole-genome alignment to generate core-genome single-nucleotide polymorphisms (SNPs) using HarvestTools Parsnp v.1.5.0 and a randomly picked genome from the collection as reference [26]. These SNPs were used to generate a maximum-likelihood phylogeny with RAxML v.8.2.11 [27], which is embedded in Parsnp, and subsequently corrected for recombination using ClonalFrameML v1.11–3 [28]. The final tree was visualized along with annotations using iTOL v.6.6 [29] and was rooted at mid-point, assuming the same evolutionary rate for all branches. For accurate SNP distance estimation between longitudinal samples, BacDist [30] was run using *E. faecium* reference genome GCF_000174395.2. Local outbreak clusters in the phylogenetic tree were defined as ≥3 isolates with <10 SNPs between the isolates and SNP distances were determined by running Parsnp within each MLST type.

## Results

### Emergence of linezolid- and vancomycin-resistant *E. faecium* isolates

**Fig. 1.**
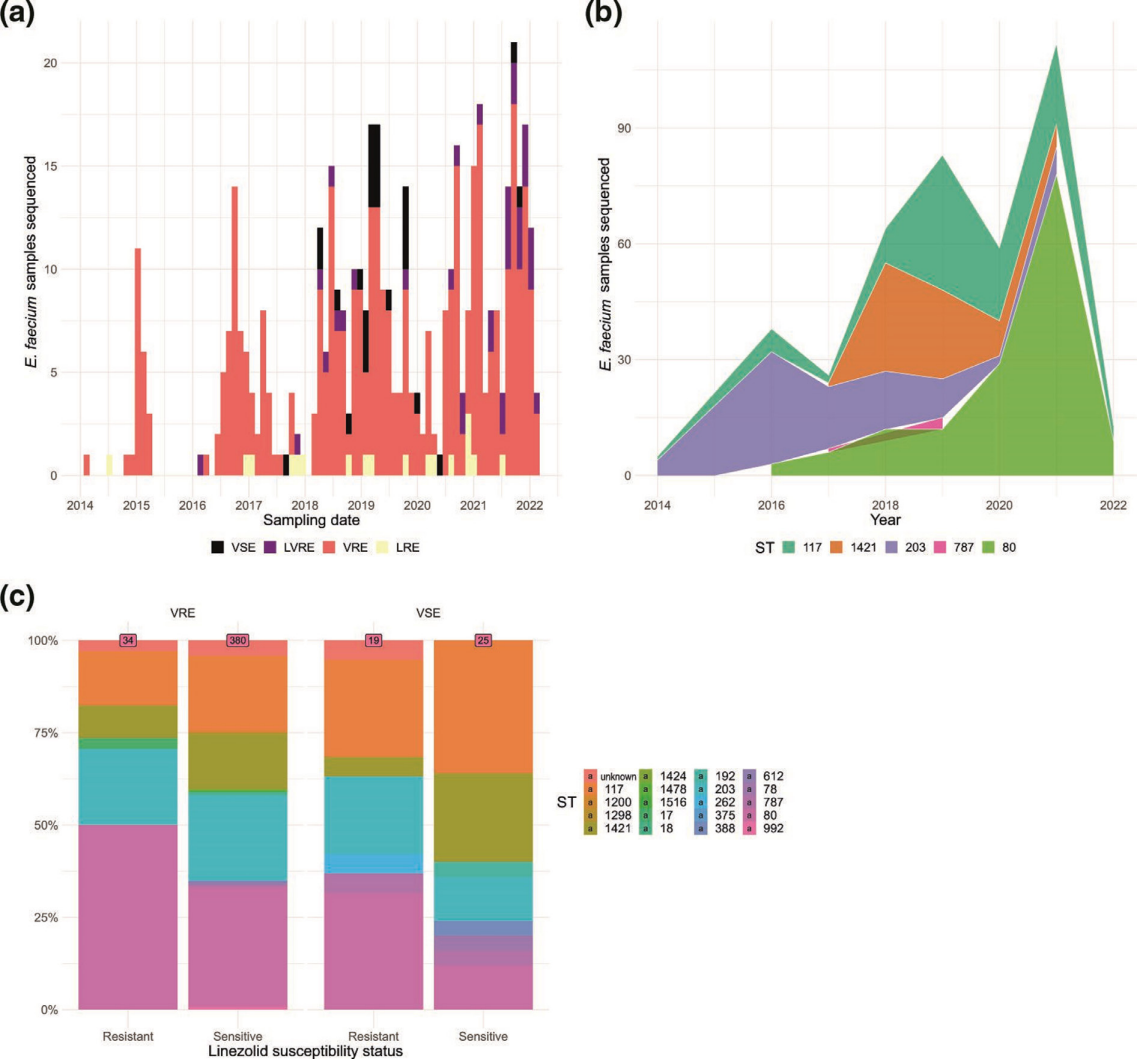
(a) Number of *E. faecium* isolates sequenced over time, colour-coded by their linezolid and vancomycin resistance genotype status from 2014–February 2022. (b) Number *of E. faecium* isolates sequenced over time depicted as an area graph. The frequency distribution of the five dominant sequence types (STs) per year is represented. (c) Relative abundance of the STs including class ‘undefined’, segregated by linezolid and vancomycin susceptibility status. Labels on top indicate the number of isolates in each subgroup.

The final set of 458 isolates belonged to 447 individual patients overall and were classified genetically as linezolid-resistant and/or vancomycin-resistant based on *in silico* resistance predictions (Fig. 1a, Table 1). Linezolid resistance often co-occurred with vancomycin resistance and there had been an increase in the prevalence of LRE over the past 8 years (Fig. 1a, Table 1). Classification of the isolates by MLST revealed a clear trend of single dominant sequence types (STs) in the various years (Fig. 1b). We observed the same MLST types between linezolid-susceptible enterococci (LSE) and LRE, with the most prevalent STs being 117, 80, 1421 and 203 (Fig. 1c). This led to further investigation of genetic relationship in phylogenetic analyses.

**Fig. 2.**
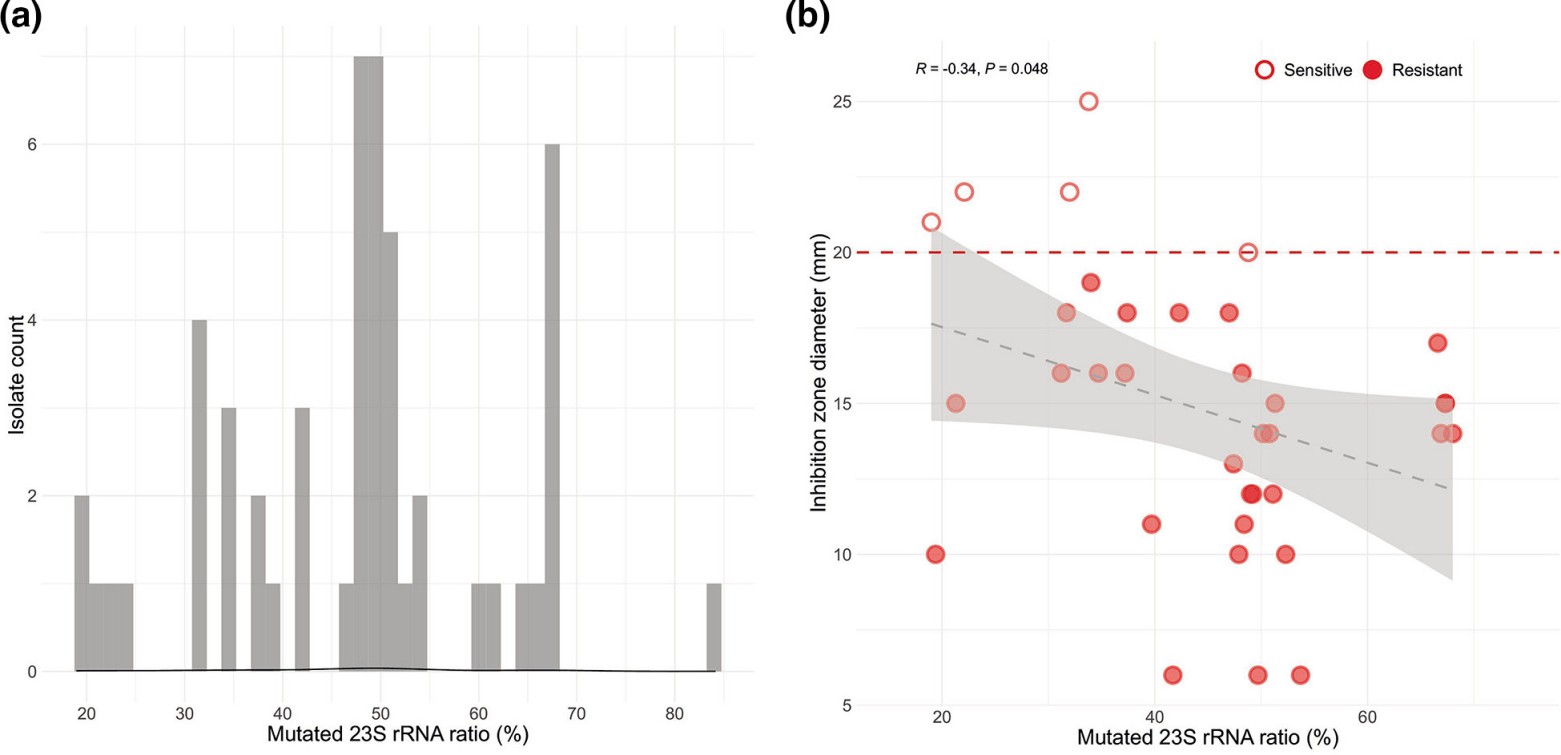
(a) 23S rRNA mutation frequency in correlation to number of sequenced isolates, as predicted by LRE-Finder. (b) Linezolid antibiotic disc diffusion zone of inhibition diameter is plotted against the predicted linezolid-mutated 23S mutation frequency. Filled circles:, isolates characterized as linezolid resistant based on agar disc diffusion; empty circles, isolates characterized as linezolid sensitive based on agar disc diffusion. Red line, EUCAST breakpoint for susceptibility. *P*=0.018, Pearson correlation, two sided-test.

We next used LRE-finder to screen the enterococcal genomes for the presence of genes known to confer resistance to linezolid [*optrA, cfr, cfr(B*) and *poxtA*] and for well-characterized resistance-conferring point mutations in the 23S rRNA gene, namely G2576T and G2505A. Fifty-two out of 53 LRE isolates carried the mutation G2576T in 23S rRNA gene. The percentage of mutated 23S rRNA reads ranged from 19–84.1 % (median 48.6 %), with between 30–70 % of reads mutated in most isolates (Fig. 2a). Two isolates carried the linezolid resistance genes *cfr* and *poxtA*, and one of these also carried the mutation G2576T. The linezolid resistant isolates included 18 LRE and 35 vancomycin-resistant, linezolid-resistant enterococci (LVRE) isolates.

### Correlation between antimicrobial susceptibility genotypes and phenotypes

We investigated whether the *in silico*-predicted resistance to linezolid correlated with linezolid resistance as measured by agar disc diffusion (Fig. 2b). The amount of mutated 23S rRNA reads was inversely correlated (R=−0.34, *P*=0.048) with the diameter of the inhibition zone. Out of 348 isolates exhibiting no mutated 23S rRNA reads, only 4 had a zone diameter <20 mm (8, 14, 17 and 19 mm, respectively), and thus, were considered phenotypically resistant without a known mechanism. Conversely, of the 35 isolates harbouring mutated 23S rRNA reads, 4 had a zone diameter >20 mm (21, 22, 22 and 25 mm, respectively), and thus, were considered phenotypically sensitive to linezolid. These four isolates had a range of mutated 23S rRNA from 19–34 %. Finally, one isolate had a zone diameter of 20 mm despite having 49 % of reads with a mutation in G2576T.

### Correlation of the occurrence of linezolid resistance with antibiotic treatment

Of the 447 patients, a total of 60 patients were treated with linezolid in the relevant period prior to sampling. Of these 60 patients, 29 (48 %) had LRE after treatment with linezolid.

**Fig. 3.**
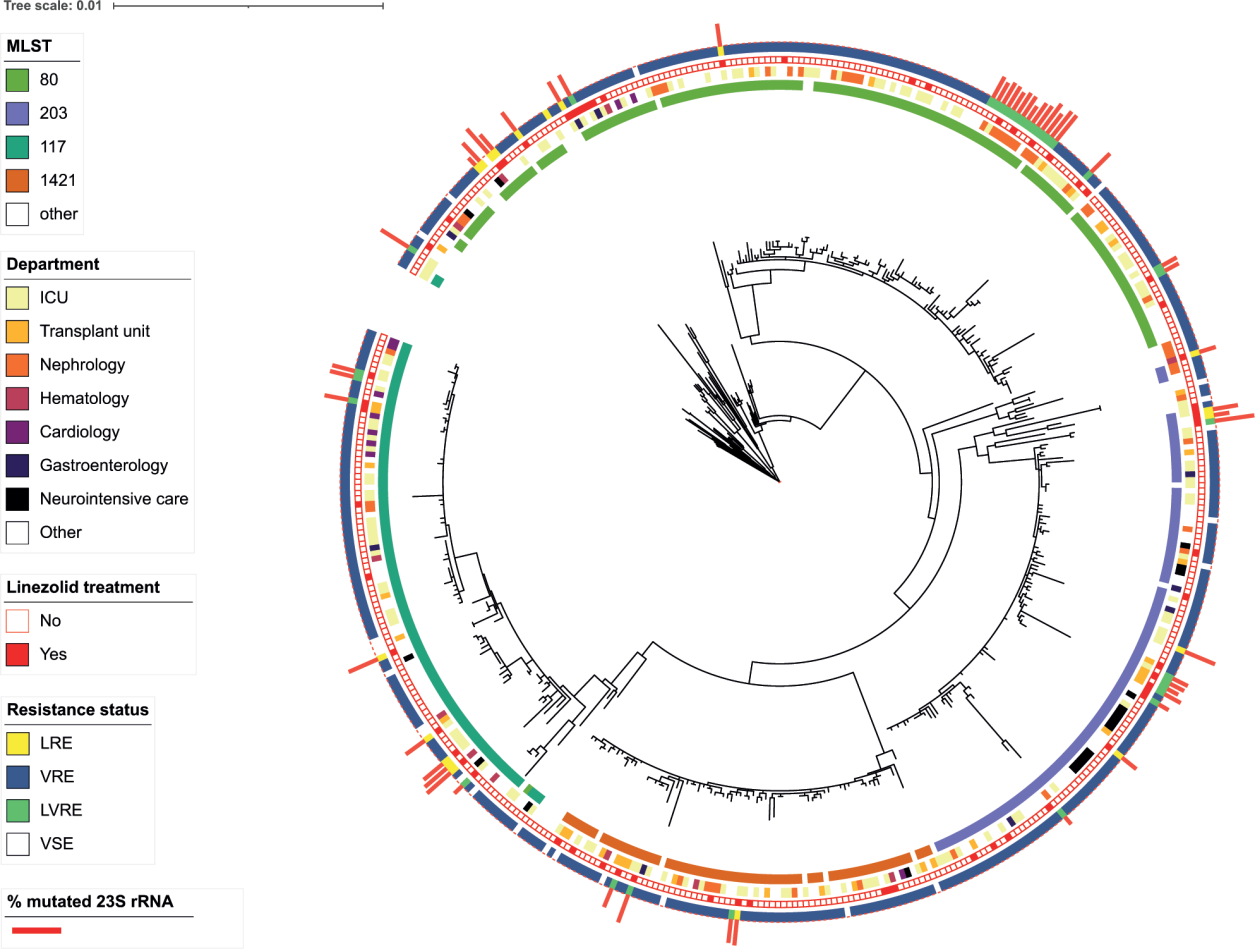
Core-genome phylogeny of all 458 *E. faecium* isolates isolated between 2014 and early 2022 at Rigshospitalet. Tree topology ignores branch length. The outer rings represent, from the inside to the outside: the MLST profiles [sequence types (STs)] of the individual isolates (white corresponds to an isolate that belongs to the less common STs or to an unclassified isolate); the wards in which patients were hospitalized; linezolid treatment of the individual patients versus no treatment; linezolid/vancomycin susceptibility status of the isolate as defined by presence of relevant mutations/resistance genes; number of mutated 23S rRNA copies.

We sought to identify whether the *E. faecium* isolates were phylogenetically closely related, possibly indicating nosocomial transmission. We constructed a phylogenetic tree with all 458 isolates to detect phylogenetically closely related isolates and possible nosocomial transmissions (Fig. 3, Table S1, available in the online version of this article). The tree contains a larger outbreak of ST80 LVRE isolates which are closely related to isolates of the same prevalent VRE clone (Fig. 3). This represents an outbreak in a particular department with 14 LVRE isolates. Notably, the occurrence of LRE isolates clustering with closely related isolates from the same department was not necessarily associated with linezolid consumption by the affected individual patients (Fig. 3). For this particular outbreak only three patients had been treated with linezolid.

For the other MLST types we also identified that some LRE/LVRE isolates were genetically closely related to outbreak clusters (Fig. 3). Specifically, of the 53 linezolid-resistant isolates, 28 were genetically closely related to other isolates in the tree (defined as clusters of <10 SNPs), and the remaining 25 isolates were not part of outbreak clusters. Of the 28 isolates with close relatives, 14 (50 %) were correlated with treatment with linezolid, while of the 25 isolates that were not part of outbreak clusters, 16 (64 %) were correlated with treatment with linezolid, leaving 9 (36 %) of these isolates that were not associated with linezolid treatment (Fisher’s exact test, *P*=0.42). We further investigated whether isolates without vancomycin-resistant genotypes were closely related to any other isolate in the tree or whether linezolid resistance for these isolates seemed to have occurred sporadically, i.e. *de novo* generation of linezolid resistance, alternatively explained by plasmid loss in highly transmissible LVRE clones. Specifically, 6/18 (33 %) LRE (without vancomycin resistance) isolates were without close relatives. Of these six isolates, four were associated with linezolid treatment of the respective patient, indicating possible *de novo* generation of linezolid resistance after linezolid treatment.

### Within–patient evolution of linezolid resistance in VRE outbreak strains

Out of the overall 447 patients, from whom an *E. faecium* was isolated between 2014 and 2022, we identified 6 patients, who were initially infected with a linezolid-susceptible *E. faecium *, but from whom a resistant isolate exhibiting the identical MLST could be isolated after linezolid therapy (Table 3). In all six cases both the sensitive and the resistant isolates were vancomycin-resistant and only exhibited small genetic distances (often <10 SNPs) to isolates from other patients, indicating that linezolid resistance possibly developed in VRE outbreak strains following linezolid treatment.

**Table 3.** Patients receiving linezolid treatment and sampled with LSE isolates prior to treatment followed by an LRE isolate after treatment. SNP distances were calculated using BacDist (see Methods)

Patient id	LSE sample		LRE sample			SNP distance (ref. genome considered)
	sample	Sampling date	sample	Sampling date	Mutated 23S ratio	
6	1929O643×6	January 2019	12K×901855	March 2019	51.2	2 (89 %)
9	x1K4849538	October 2018	17xK411418	November 2018	41.7	1 (84.1 %)
39	3T×2388061	August 2020	920718xE92	August 2020	48.2	2 (89 %)
17	x1738D4791	August 2018	26×84410K2	August 2018	61.4	0 (84.1 %)
13	12621×27T3	January 2022	1×2×2422D317	January 2022	50.2	1 (89 %)
10	T57×417850	November 2018	19 402D×6	October 2019	22.1	4 (89.2 %)

*Different MLST type of resistant isolate compared to first isolate.

## Discussion

In this study, we analysed the emergence of linezolid resistance in a large collection of *E. faecium* isolates recovered from a tertiary hospital over more than 8 years. We combined information on antibiotic treatment and patient localization in the hospital with whole-genome sequencing data from 458 *E. faecium* isolates. Linezolid consumption has increased 1.5-fold between 2010–2019 at Rigshospitalet and the Capital Region accounts for 73 % of the overall linezolid consumption in Denmark, which is likely due to the increasing prevalence of VRE [7, 8].

Linezolid is the treatment of choice for VRE infections. In addition, we found that although linezolid resistance appeared to be transmitted in the wake of highly transmissible VRE, linezolid resistance also developed in non-outbreak situations as a consequence of linezolid treatment in individual patients. A major concern is that VRE outbreaks turn into LVRE outbreaks, highlighting the need for genomic surveillance and the monitoring and management of hospital outbreaks. The phylogenetic tree illustrates that linezolid resistance occurs in VRE isolates as well as VSE isolates. When VRE outbreaks are detected screening procedures are initiated at the specific wards, initially screening co-patients and secondarily the whole department. This is repeated every week until no VRE are detected in the department. These screening procedures likely resulted in an overrepresentation of VRE associated with an outbreak situation in our isolate collection.

The present study showed evidence of both *de novo* generation of linezolid-resistant isolates following linezolid treatment as well as dissemination of LVRE isolates in hospital wards as part of outbreaks. The consecutive patient isolates were only identified in VRE isolates in the present study due the sampling method, where we have systematically screened patients in departments with VRE outbreaks. This is a limitation of the current study.

In this study, whole-genome sequencing furthermore enabled linezolid resistance phenotype–genotype correlation studies on the presence of genetic resistance conferring markers and a linezolid resistance phenotype. The identified mechanisms of 23S rRNA mutation in this study has previously been described as a primary mechanism in other studies of *E. faecium* [10]. We did not identify the horizontally transferable mechanisms of linezolid resistance apart from a single case.

We identified a correlation between phenotypic linezolid susceptibility and a corresponding genotype, as others have previously described [31]: the number of mutated 23S rRNA copies was inversely correlated with the antibiotic disc diffusion zone diameter. This finding is in line with earlier reports on the level of resistance to linezolid that correlated with the proportion of mutated 23S rRNA copies (gene dosage effect) [32, 33]. Since 23S mutations can occur via a homologous recombination process [34, 35], linezolid treatment of patients harbouring *E. faecium* isolates with only few mutated 23S might result in higher linezolid resistance levels by facilitating an increase of mutated 23S rRNA copies. Other studies have described a larger discrepancy between the linezolid susceptibility phenotype and the genotype, indicating that novel unknown markers of linezolid resistance are likely to be described in the future [36]. Interestingly, in the few isolates of the present study with a discrepancy between genotype and phenotype, the zone diameter was often close to the breakpoint, indicating the inaccuracy of the phenotypic assay. Because homologous recombination events can rapidly increase resistance levels, genomics might in the future guide early treatment interventions by identifying isolates with a low percentage of mutated 23S rRNA reads early on. The fact that we did not find isolates where 100 % of the 23S rRNA reads harboured the resistance-conferring mutation might indicate an association of resistance-conferring mutations with increased fitness costs. The most common mutation frequency was 40–50 %. One explanation for this observation could be that the inhibition zone is often larger for isolates with lower mutation frequency (Fig. 2b), and hence, there is a greater possibility of missing isolates with lower mutation frequency in the laboratory.

The genotypes identified in this study are similar to what has been described in previous studies from the Capital Region [3, 5, 6], illustrating that linezolid resistance is likely to occur in isolates belonging to the same MLST types as VRE. The current dataset illustrates that genotypic characterization of both linezolid and vancomycin resistance is important and that this could potentially guide treatment in the future. Vancomycin- and linezolid-susceptible isolates can be treated with vancomycin. VRE isolates with *vanB* may be treated with teicoplanin in monotherapy or linezolid as monotherapy. Rarely, a combination of linezolid and daptomycin is administered. However, for LVRE isolates there are no oral treatment options, which is why intravenous administration of antibiotics is necessary. Patients require i.v. catethers and close contact with the hospital. This study exemplifies that high consumption of linezolid can result in the emergence of LRE. This highlights the need for rational consumption of antibiotics, including linezolid, and illustrates the need for studies of linezolid treatment.

In summary, the present study demonstrates that linezolid-resistant *E. faecium* arise both in individual patients after linezolid exposure and due to transmission in the hospital setting, highlighting the need for genome-based surveillance of LRE in order to secure rational treatment with linezolid.

## Supplementary Data

Supplementary material 1
